# Management of Fournier’s gangrene with skin grafting by bagging technique of testes: case report

**DOI:** 10.3205/iprs000128

**Published:** 2019-02-04

**Authors:** Yasir Ali AlShehri, Hiba AlBurshaid, Layan AlBassam, Khalid AlMutairi

**Affiliations:** 1 Department of Surgery, College of Medicine, Imam Abdulrahman Bin Faisal University, Dammam, Saudi Arabia; 2 Department of Plastic Surgery and Burn Unit, King Fahad Military Medical Complex, Dammam, Saudi Arabia

**Keywords:** fournier, bagging technique, testes, gangrene, skin graft

## Abstract

**Aim:** To share our experience with the management of Fournier gangrene (FG) using the bagging technique of the testes, and to highlight the importance of implementing a multidisciplinary approach in managing FG.

**Case** **presentation:** A 58-year-old male with type 2 diabetes mellitus (DM) was brought to the emergency department (ED) with necrotizing fasciitis involving the genitalia; he was managed in the ED with Intravenous (IV) fluid resuscitation and IV antibiotics. The surgical team was consulted and multiple debridement procedures were done. Healthy granulation tissue was formed within one month of the serial debridement. A split-thickness skin graft using bagging technique of the testes and vacuum-assisted closure (VAC) were applied. The patient was reassessed one year following presentation, and a result with a near normal appearance was achieved with complete preservation of functional outcome.

**Conclusion:** FG is a type of necrotizing fasciitis that could be managed either conservatively with IV antibiotics and/or hyperbaric oxygen, or surgically by debridement and applying VAC.

In our case, the testes were debrided and bagging technique of the testes was used. It’s believed that with this technique, the overall cosmetic and functional results are superior.

## Introduction

Necrotizing fasciitis (NF) is a life-threatening condition that’s caused by toxin-producing bacteria and affects the fascia [1]. One type of NF is Fournier gangrene, which mainly involves the inguinal and genital area [2]. Jean Fournier, a dermatologist and venereologist, first described FG clinically in the late 1800s. He described it as an idiopathic fulminant gangrene of males’ inguinal and genital region that occurs rapidly and progressively [3]. This rare and fatal infection, which mostly affects immunocompromised individuals, is a result of both aerobic and anaerobic bacteria [2], [3]. Yilmazlar et al. found that Escherichia Coli was the most commonly identified microorganism in these cases [4]. Early diagnosis, antibiotic treatment, surgical debridement and reconstruction of the affected area are the main aspects of managing this condition [5]. In addition to these main steps, bagging technique of the testes was done, in which both testes were covered together with a split-thickness skin graft. With this technique, a near normal appearance and a complete functional outcome were achieved. 

## Case presentation

A 58-year-old male who is known to have type 2 diabetes mellitus (DM) presented to the emergency department (ED) with fever, vomiting, scrotal pain and swelling. After being thoroughly evaluated in the ED, the patient was clinically diagnosed with Fournier’s gangrene. Intravenous (IV) fluid resuscitation was initiated, IV antibiotics were given, and the surgical team (including urology and plastic surgery) was consulted.

**Figure 1:**
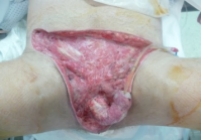
Necrotic tissue of the genitalia was extensively debrided.

**Figure 2:**
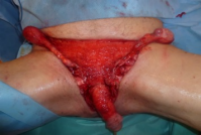
Testes were relocated in the anteromedial side of the thighs. Granulation tissue has been formed.

The patient was shifted to the operating room (OR) where debridement was done by urology team (Figure 1 (Fig. 1)). The testes were temporarily relocated to the anteromedial side of the thighs in order to achieve an optimum scrotal wound closure (Figure 2 (Fig. 2)).

**Figure 3:**
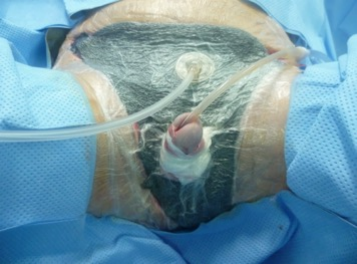
VAC was applied to aid formation of granulation tissue.

Following that, he underwent surgical debridement of the scrotum and penis four times, and vacuum-assisted closure (VAC) was applied to enhance wound healing (Figure 3 (Fig. 3)).

**Figure 4:**
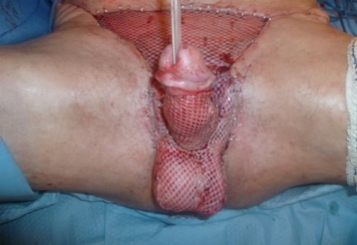
Testes were approximated and the bagging technique was done using the skin graft.

A healthy granulation tissue was formed over the wound within one month of serial debridement. Repositioning of the testes to their natural position was done and they were both sutured together by absorbable sutures. A split-thickness skin graft was used in three units to reconstruct the defect in the penis and inguinal area. The bagging technique was done by harvesting the skin from the back and was applied from the base of the penis and brought into the frontal part of it. The testes were approximated at the midline and were attached together with small sutures (Figure 4 (Fig. 4)).

**Figure 5:**
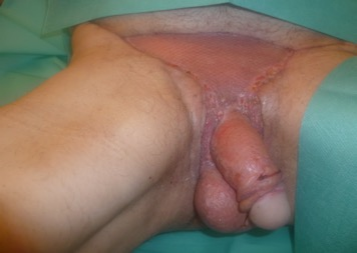
Satisfactory healing and cosmetically near normal appearance was achieved after two months.

After that, a mini abdominoplasty was done to the patient for a more enhanced shape and satisfactory result. A near normal appearance and satisfactory result was achieved after two months and patient was discharged home with good urological and sexual function (Figure 5 (Fig. 5)). The patient was reassessed one year following the surgery and was satisfied and with no complications.

## Discussion

FG is known to be a type of necrotizing fasciitis that affects the external genitalia, including the penis and scrotum of immunocompromised individuals [5], [6].

FG has many predisposing factors, the most common one being diabetes mellitus with an incidence of 46–76.9% [7]. Other factors that may contribute to the condition include alcoholism, malignancy, obesity and extremes of age [5], [6].

Fournier’s gangrene is believed to be polymicrobial in origin, caused by both aerobic and anaerobic organisms that invade the area through skin abrasions caused by trauma or ulceration. This, along with the favorable environment for bacteria to grow that is found in high-risk individuals all contribute to the emergence of the disease [8].

In the majority of reported cases, there are underlying causes that may trigger FG including anal abscesses or trauma caused by a certain medical interventions to the scrotum or perineum [9]. However, in our case no cause was found. 

Despite advances in the medical field, the mortality rate of FG remains as high as 67%; thus, proper management steps should be carefully followed [7]. Early recognition and diagnosis, aggressive antibiotic administration, fluid resuscitation, extensive surgical debridement are all important determinants of the outcome [3], [7]. Today, the gold standard therapy for fournier’s gangrene is early and complete surgical debridement [10]. Defects where large areas of tissue are lost necessitate reconstruction of the region by skin grafts or flaps [5]. Other treatment modalities include hyperbaric oxygen (HBO) therapy [11], which is a widely accepted method that may help in the management of severe cases. Wound healing may be accelerated by using urinary and fecal diversion techniques [12].

## Conclusion

Fournier’s gangrene is known to be a surgical emergency [13]. One of the important risk factors leading to this condition is Diabetes Mellitus [14]. Management of FG includes administration of IV antibiotics, fluid resuscitation and prompt surgical debridement and reconstruction by either skin flaps or grafts [15]. Hyperbaric oxygen (HBO) therapy [11] has proven beneficial in managing severe cases. Acceleration of wound healing may be achieved by diversion of urine and feces away from the affected area [12]. In our case, skin grafts were applied using the bagging technique of the testes, which resulted in a satisfactory cosmetic and functional result.

## Notes

### Competing interests

The authors declare that they have no competing interests.

### Ethical statement

- Ethical approval: Ethical approval for this study was obtained from the responsible ethics committee.
- Informed consent: Informed consent was obtained from all individual participants included in the study.
